# No seasonal variation in physical activity of Han Chinese living in Beijing

**DOI:** 10.1186/s12966-017-0503-1

**Published:** 2017-04-17

**Authors:** Guanlin Wang, Baoguo Li, Xueying Zhang, Chaoqun Niu, Jianbo Li, Li Li, John R. Speakman

**Affiliations:** 1 State Key Laboratory of Molecular Developmental Biology, Institute of Genetics and Developmental Biology, Chinese Academy of Sciences, Beijing, China; 2 University of Chinese Academy of Sciences, Beijing, China; 3 Department of Physiology and Pathophysiology, Basic Medical College of Dali University, Dali, Yunnan China; 4 Institute of Biological and Environmental Sciences, University of Aberdeen, Aberdeen, UK

**Keywords:** Physical activity, Obesity, Environment, Seasonal variation, Han Chinese, Temperature, PM2.5, Intensity

## Abstract

**Background:**

Physical activity (PA) is widely acknowledged to be beneficial to health and wellbeing, and is potentially influenced by a variety of environmental factors such as ambient temperature, weather conditions and air pollution levels. Since these factors vary seasonally, physical activity participation may also respond seasonally. Current population studies to profile physical activity often sample individuals only once, and this may result in biased estimates if there is strong seasonal variation.

**Methods:**

We conducted a study of 40 Han Chinese adults living in Beijing using GT3X accelerometers. We measured PA levels every two months across a complete year, while simultaneously monitoring ambient temperatures and air pollution levels. Average hourly vector magnitude (VM) and percentage time spent at each PA intensity (sedentary to light, moderate, vigorous and very vigorous) were measured. General Linear models (GLMs) were used to explore the effects of time of day, temperature and PM 2.5 levels on PA. One way ANOVA was used to test whether there were seasonal differences in body weight and body fatness.

**Results:**

The main factors influencing activity levels were the time of day and individual characteristics including age and body fatness, but there was no significant difference between the months. In addition, there was no significant impact of either ambient temperature or air pollution levels (PM2.5). There were also no significant differences over the year in the time spent at sedentary-light, moderate and very vigorous PA levels, but for vigorous PA level which occupied less than 0.5% daily physical activity, both month and individual were significant factors.

**Conclusions:**

The relatively constant pattern of urban daily life, independent of time of year, may override the potential impacts of environmental factors that would be anticipated to impact PA levels. These subjects did not specifically avoid activity coincident with elevated air pollution levels (PM2.5). Single week long measurements of physical activity could provide a representative measurement of the physical active levels in this population.

**Electronic supplementary material:**

The online version of this article (doi:10.1186/s12966-017-0503-1) contains supplementary material, which is available to authorized users.

## Background

Physical inactivity has become a leading risk of non-communicable diseases including cardiovascular disease, type 2 diabetes mellitus (T2D) and cancer [1, 2]. Participation in at least 150 min per week of moderate to vigorous physical activity (MVPA) has been reported to decrease blood pressure, alleviate type 2 diabetes (T2D) and reduce the risk of cancer [1, 3–5], and was therefore recommended by the WHO as a target level of physical activity for adults aged 18–64 [1]. However, it was estimated that globally around 23% of adults (20% of males and 27% of females) aged 18 and over did not meet these guideline levels in 2010 [1].

Although several studies have suggested that there is a genetic contribution to the differences of PA levels [6–9], it is generally assumed that participation in PA is mostly under voluntary control, and it is believed that environmental conditions exert either a facilitating or constraining influence on such voluntary participation [10]. Such factors include variation in ambient temperature, because individuals may avoid exercise when it is too hot because of the risk of hyperthermia [11] and when it is subzero because of the possible risk of falls and injury during activity, due to ice formation. Other weather conditions, such as strong wind, precipitation and/or other extreme weathers may also influence activity levels [10]. The duration of daytime may also be an important factor for both convenience and safety considerations of being outside during the period of darkness [10]. All of these factors vary seasonally and to different extents in different places, hence it is expected that PA levels may also vary temporally and spatially. This may then have a large impact on the spatial and temporal patterns of disease risk [12, 13]. In the USA, for example the prevalence of type 2 diabetes is increased in areas with higher average annual temperatures [12], which could in theory be contributed to by people avoiding activity at higher temperatures. It has been previously demonstrated that seasonal PA changes coincided with seasonal changes in blood pressure, body mass and affective disorders [14, 15].

Air quality, especially PM 2.5 (the levels of particulate matter with a diameter less than 2.5 microns), is a major public health issue in many large cities [16–18]. High levels of PM2.5 have been shown to be detrimental to health, causing respiratory disorders and severe lung dysfunction, along with elevated risks of lung cancer and cardiovascular events [19, 20]. It seems likely that the health benefits of PA may be negated at high levels of air pollution [18]. However, the extent to which urban individuals avoid being active when PM2.5 levels are high is currently unknown. PM2.5 also varies seasonally [21], leading to an additional potential seasonal impact on both PA and health [22].

Several different approaches are available for quantification of PA levels, from subjective measurements like self-report questionnaires [23–25], to various forms of direct assessment, including doubly-labeled water [26], pedometers [27], heart rate monitors (HRMs) [28] and accelerometers [29]. Tri-axial accelerometers are small monitors that record movements of the body in three dimensions and have hence emerged as a popular method for objectively monitoring PA levels [30]. Among such accelerometers, the GT3X (ActiGraph, Inc. Pensacola FL, USA) is a particularly popular device to explore objective patterns of PA in terms of the elemental characteristics such as intensity, duration and frequency [31]. The GT3X has been used in a large number of studies including the National Health Assessment in the USA (NHANES) [32].

Almost all population based studies of PA have used single measurements, averaged over a few adjacent days, to profile the PA levels. It is unclear whether single measurements provide an adequate representation of PA. For example, there could be large biases if there were large seasonal differences in PA. This is especially the case for meta-analyses or comparison studies where the differences due to seasonal effects may override other effects, or generate spurious differences between populations.

Previous studies of adults in the UK [33–36], US [14, 37] Netherlands [38] and Canada [39] all showed seasonal variation in physical activity, with higher PA levels observed in summer compared with winter, using both self-report assessments or pedometers or doubly-labled water and accelerometers to record PA. Similar results were also shown in studies on seasonal variations in children (aged 8–11) in Denmark [40]. A review by Rich [41] also reported that overall PA of children living in UK is lower during winter. In Norway, 9-years-old children had significantly higher mean physical activity levels in spring than in winter and fall, when measured using a uni-axial MTI Actigraph accelerometer (Model: 7164), however, no associations were found between mean physical activity level and season among 15-year-olds in Norway [42]. Contrasting these studies showing higher activity in summer, a study using GT3X monitoring of the PA of Australian children suggested that PA decreased in summer compared to winter [43]. These differences potentially reflect the prevailing ambient temperature regime in the respective locations–in Melbourne, Australia at 37 ^o^S it may be too hot to exercise in summer, while in Norway at 57–72 ^o^N it may be too cold to exercise in winter. To our knowledge, no published studies of seasonal variation in PA have been performed in Han Chinese populations.

Beijing, the capital city of China, at 40 ^o^N is profoundly seasonal in its climate, with hot summers that have ambient temperatures routinely over 40 °C and cold winters, with ambient temperatures often falling to −10 °C. Most precipitation falls in the summer. In addition, it has high and extremely variable levels of air pollution (PM2.5) which can vary from an air quality index (AQI) of 10 to over 600. In the present study we recruited 40 Han Chinese adults living in Beijing (defined as living inside the 6^th^ ring road) to examine (1) whether there was a seasonal effect on the average hourly PA measured over 7 continuous days; (2) whether the ambient temperature and PM2.5 influenced the intensity and duration of the PA; (3) whether there was an overall seasonal effect on different intensities of activity.

## Methods

### Subjects

Forty volunteers (19 females and 21 males) were recruited by posters near Olympic Park in Chaoyang District, Beijing. Of the 40 volunteers, 18 were Master/Ph.D students of University of Chinese Academy of Sciences located next to the park, the rest had various occupations, but all were in employment. At the end of 1 year, 37 individuals had completed the study (3 women dropped out due to pregnancy). Data from 34 individuals (13 female and 21 male, aged 31 ± 10) were used for final analysis (provided valid data for at least 4 days in each measurement period, study flowchart, Additional file 1: Figure S1). The subjects were asked to visit our lab every 2 months to get a GT3X device fitted and had their body composition measured using TANITA ® TBF-418B. During their first visit they additionally had their height, weight, waist and hip circumference, body composition, blood pressure, heart rate measured and they completed a life-style questionnaire (see in Additional file 1). Subject characteristics are in Table 1.

**Table 1:** Baseline of demographic and anthropometric variables in 34 subjects (involved in final data processing

	Total (Mean ± SD)	Female (Mean ± SD)	Male (Mean ± SD)
Age (years)	31 ± 10	33 ± 12	29 ± 8
Weight (kg)	66.3 ± 8.6	61.1 ± 6.7	69.5 ± 8.2
BMI (kg/m2)	23.1 ± 2.3	23.0 ± 2.2	23.2 ± 2.4
% BF	23.2 ± 7.7	31.3 ± 3.8	18.3 ± 4.5
FM (kg)	15.4 ± 5.5	19.3 ± 4.4	12.9 ± 4.6
FFM (kg)	50.9 ± 8.4	41.8 ± 2.8	56.6 ± 4.8
WC (cm)	83.1 ± 7.7	78.8 ± 6.0	85.7 ± 7.5
HC (cm)	99.5 ± 4.4	100.0 ± 4.4	99.1 ± 4.5
SBP	124 ± 16	115 ± 18	129 ± 12
DBP	75 ± 11	73 ± 13	77 ± 9
HR	74 ± 9	71 ± 7	76 ± 10

*BMI* body mass index, *% BF* body fat percentage, *FM* fat mass, *FFM* fat free mass, *WC* waist circumference, *HC* hip circumference, *SBP* systolic blood pressure, *DBP* diastolic blood pressure, *HR* heart rate

### Physical activity measurements

The subjects were instructed to wear a GT3X monitor on a waist band for 24 h a day (including sleeping time) over 7 continuous days and to remove the monitor only during showers, bathing, swimming or other water activities. Activity loggers were fitted to each individual every 2 months, i.e., in January, March, May, July, September and November in 2015 respectively (see in Additional file 1). The recording epoch was set to record by minute. Hourly physical activity was summed using the minutes’ VM (vector magnitude) data within an hour (here we defined as from 0:00 to 0:59 as 1 h). Then we calculated average hourly PA across days when the monitor was worn called ‘worn days’ (at least 4 worn days was regarded as a valid sample) of each subject in each month. Percentage time spent in different physical activity (PA) intensity levels (sedentary to light, moderate, vigorous and very vigorous) of the worn time was used to examine the difference of seasonal variability.

### Temperature and PM 2.5 data Acquisition

Hourly temperature and PM 2.5 data was obtained from the Beijing Urban Ecosystem Research Station. The data collection site is at 40.00729 ^o^N, 116.3374 ^o^E. This is around 6 km from the general area where the subjects were recruited. The data paired with physical activity period was selected for analysis.

### Statistics

Minitab 16 was used to analyze the data. We used one-way ANOVA to compare whether there was a change in body composition between months. General linear models (GLM) were used to analyze the temperature differences between months and the Tukey method (95% confidence) was used for pairwise comparison by month. Month, time and month*time were set as factors. We also used GLM to examine the PM2.5 difference using month, time and month*time as factors with temperature as a covariate. Tukey tests (95% confidence) were used for pairwise comparison by month. Mixed models were used to analyze the difference in average hourly PA levels (mean hourly VM), we used month (the seasonal factor, fixed), time of day (fixed), ID (random), month*time, month*ID and time*ID as factors, with simultaneous hourly ambient temperature data (°C) and air quality data (PM 2.5) as covariates. None of the interactions were significant and were eliminated. For physical activity intensity comparison, we used percentage of total physical activity as response and included month and ID as factors and temperature and PM2.5 as covariates. Further individual characteristics analysis were used GLM including month as factor which covaried with age and body fatness (body fat percentage).

## Results

### Temperature and air quality (PM2.5) varied seasonally

**Fig. 1:**
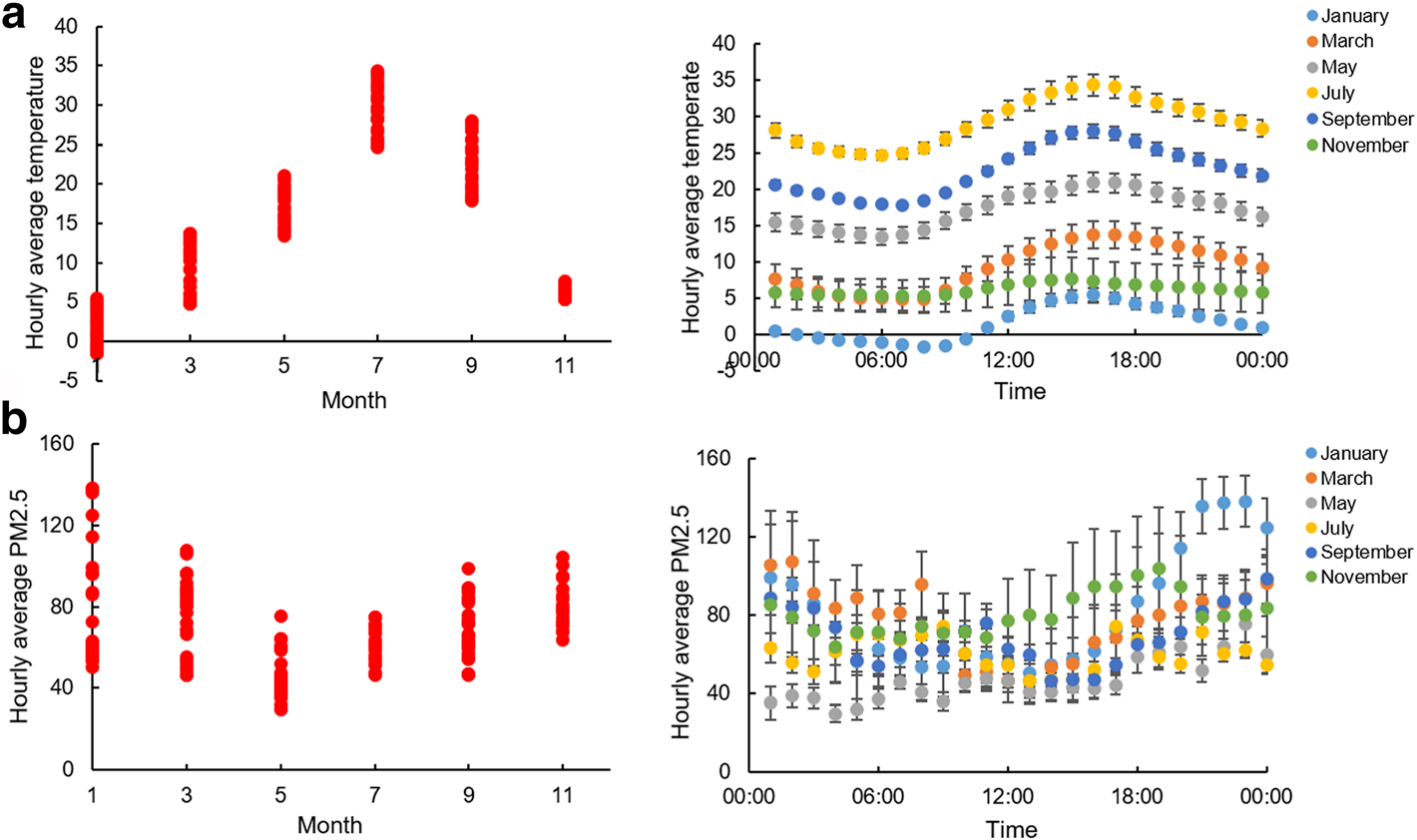
Seasonal variation of temperature and PM2.5. **a** shows the monthly difference of temperature and hourly average temperature range from January, March, May, July, September and November across the complete year (2015). There was a significant difference of temperature monthly and hourly. **b** shows the monthly difference of PM2.5 and hourly average PM2.5 during the six measured months. There was a significant difference of PM2.5 monthly and hourly which also covaried with temperature

Hourly temperature (°C) and air quality (PM2.5) data for the days when subjects were wearing activity loggers were retrieved and compared by month. There were clear seasonal variations of both temperature and PM2.5. Month, time of day and the interaction between month and time of day all had significant effects on the ambient temperature (Fig. 1a, F_(5, 4512) month_ = 36377.29, *P* < 0.001; F _(23, 4512) time of day_ = 550.81, *P* < 0.001; F _(115, 4512) month*time of day_ = 15.85, *P* < 0.001). In July, the average temperature was 29.3 °C while in January it was 1.6 °C. PM 2.5 level also varied by different seasons. Month, time of day and month*time of day as well as ambient temperature were all significant factors influencing the PM2.5 levels (Fig. 1b, F_(5, 4511) month_ = 544.09, *P* < 0.001; F _(23, 4512) time of day_ = 141.12, *P* < 0.001; F _(115, 4511) month*time of day_ = 38.43, *P* < 0.001, F _(1, 4512) temperature_ = 603.95, *P* < 0.001). In May, the average PM 2.5 was 47, which was significantly lower than in January when it averaged 81 and November when it was on average 80.

### No seasonal body composition change

**Fig. 2:**
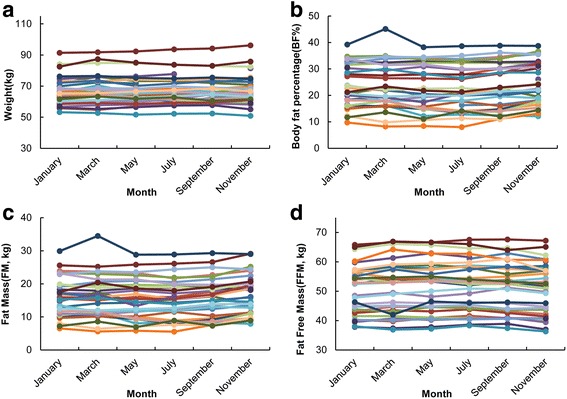
Seasonal difference in body composition. **a** is the seasonal difference in body weight. Each point represents an individual. **b** represents the seasonal difference in body fat percentage. **c** shows the seasonal difference of fat mass. **d** shows the seasonal difference of fat free mass. There was no significant effect of season for any of the traits

Subjects were measured for basic demographic and anthropometric data before the first measurement period (Table 1). Body composition was measured on each occasion that the GT3X was worn. Over the following year there was no significant effect of month on body weight (kg) (Fig. 2, one-way ANOVA: F_(5,190)_ =0.06, *P* = 0.998), body fat percentage (%BF) (Fig. 2, one-way ANOVA: F_(5,190)_ =0.3, *P* = 0.912), fat mass (FM, kg) (Fig. 2, one way ANOVA: F_(5,190)_ =0.32, *P* = 0.900) and fat free mass (FFM, kg) (Fig. 2, one way ANOVA: F_(5,190)_ =0.08, *P* = 0.995).

### No seasonal effect on average hourly PA

**Fig. 3:**
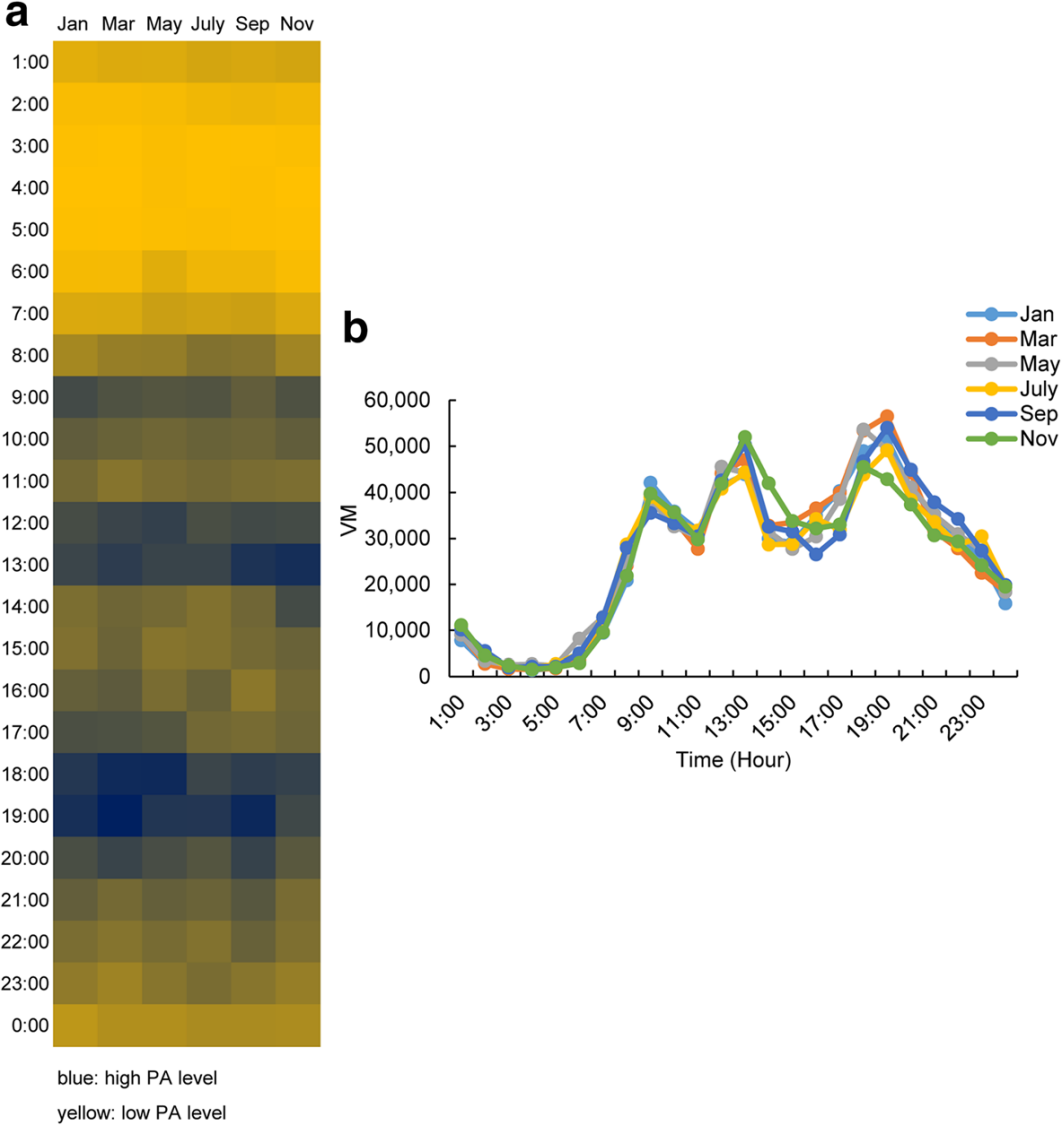
Profile of 24-h total physical activity of each month. **a** is the heatmap of physical activity profile where darker blue indicates the higher PA level while the lighter yellow represents the lower level. The most active times of a day were around 9:00, 12:00–13:00, 18:00–19:00, either the time after meals or commuting between work and home. **b** represents the level of physical activity level across the 24 h in a day. There is no significant difference of the average physical activity across the months

We calculated the average hourly vector magnitude (VM) for each hour over the whole 7 day period of measurement of each subject every 2 months (Table 2). The patterns of average hourly physical activity measurement are illustrated in Fig. 3. In Fig. 3a, the darker blue indicates higher physical activity. Periods of high PA occurred at around 9:00, 13:00 and 18:00–19:00. The highest VM level was around 50,000 counts/h (Fig. 3b). We found both individual ID (F _(33,4567)_ =16.05, *P* < 0.001) and time of day (F_(23,4567)_ =105.37, *P* < 0.001) were significantly associated with PA, however, month was not a significant factor (F _(5,4567)_ =0.64, *P* = 0.668). Although there were significant seasonal differences in both ambient temperature and PM2.5 (Fig. 1), neither temperature (F_(1,4567)_ =2.54, *P* = 0.111) nor PM2.5 (F_(1,4567)_ =0.04, *P* = 0.836) had significant effects on the average hourly PA. The absence of an effect of these factors could be because each day included a significant portion of time when subjects were inactive because they were sleeping, and this day to nighttime difference dominated any other patterns of variation. To explore this possibility we analyzed the data collected between 9:00 and 23:00 to remove the sleeping time. The results were similar to those collected when the whole day was included in the analysis, with time of day and ID the most significant factors (F_(5,2854)__month_ = 1.28, *P* = 0.268; F_(14,2854)__time_ = 30.7, *P* < 0.001; F_(33,2854)__ID_ =16.2, *P* < 0.001) but no significant effects of temperature or PM2.5 (F_(1,2854)__temperature_ = 3.18, *P* = 0.074, F_(1,2854)__PM2.5_ = 0, *P* = 0.986). As ID was an important indicator, we also calculated the average daily PA as a response variable, with month, age and average body fatness to establish the characteristics of individuals that influence their PA. Both fatness and age were significantly associated with the PA level (F_(1,191) age_ = 5.74, P = 0.017, F_(1,191) body fatness_ = 6.49, *P* = 0.012).There was a negative relationship between body fatness and the PA level (t = − 2.55, *P* = 0.012) indicating that individuals with higher body fatness level were less active than those with lower body fatness. We also found that there was a positive relationship between age group and activity, indicating that older people were more active than younger ones in our volunteer group (t = 2.40, *P* = 0.017).

**Table 2:** Average hourly vector magnitude (VM) for each hour of every 2 months

Time	January		March		May		July		September		November	
	mean	SD	mean	SD	mean	SD	mean	SD	mean	SD	mean	SD
01:00:00	7831	8336	9068	9862	8993	11871	10927	10702	10165	7006	11171	12113
02:00:00	2802	3332	2823	2739	3427	3442	4799	4768	5503	9579	4558	4803
03:00:00	2066	1647	1680	1490	2554	3635	1973	1418	2009	1855	2384	2229
04:00:00	1420	749	1573	997	2700	3835	1953	1653	2159	2026	1557	1127
05:00:00	1872	1452	1779	1167	2362	2470	2663	2568	2161	1949	1995	1215
06:00:00	3586	6220	3777	9433	8211	22993	4931	9385	5029	7691	2912	3778
07:00:00	9455	15188	9681	13423	12937	21180	11864	16728	12816	14096	9608	12958
08:00:00	20873	18247	24149	18282	24675	20639	28698	24599	27964	24561	21916	24855
09:00:00	42184	25712	39588	25187	38419	19582	39049	22611	35537	21203	39760	28426
10:00:00	35910	20961	34122	18261	32597	12718	34102	11699	33315	12061	35692	13651
11:00:00	31767	18405	27697	19375	30349	16666	31732	13350	30495	12069	29788	13300
12:00:00	41435	16396	44139	16022	45558	19140	40854	18785	42674	14120	41905	16227
13:00:00	43968	22009	46963	26146	44274	27049	44295	32324	50311	31082	52027	32190
14:00:00	29995	14240	32829	20081	31548	12555	28650	16233	32528	12388	42024	16935
15:00:00	29038	13949	33505	17543	27704	10697	28803	16407	31464	13853	33841	17615
16:00:00	34924	26957	36603	21291	30419	11419	34266	15982	26559	11839	32181	14114
17:00:00	40352	36797	39954	24163	38600	17657	31941	14782	30843	9660	33069	16182
18:00:00	48932	26282	53389	26688	53635	20178	43934	20554	46715	20282	45489	17540
19:00:00	51556	18264	56512	24806	49271	15903	49066	17664	54047	30909	42926	18382
20:00:00	40837	19268	44352	27134	41169	21697	38385	17214	44948	27716	37415	20501
21:00:00	35284	24434	31589	24902	35098	19656	33633	17563	37905	26054	30694	19646
22:00:00	30293	18278	27841	24975	30998	23039	28539	17309	34276	25341	29346	15936
23:00:00	25611	17222	22494	17917	27420	20864	30471	15741	27355	17910	24169	11991
00:00:00	15854	10915	18433	15171	18353	14281	19825	10849	19885	16433	19503	13609

### No seasonal effect on physical activity intensity

**Fig. 4:**
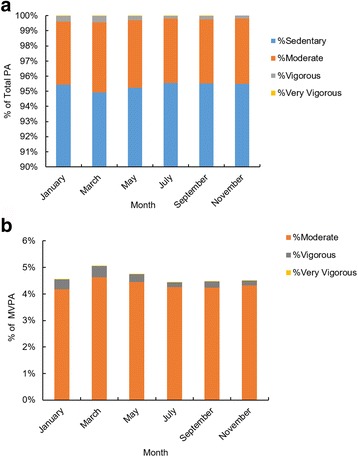
Seasonal differences in the intensity of physical activity. **a** is the patterns for percentage time spent at each intensity level of physical activity in each month. The sedentary to light physical activity always occupied almost 95% of the time. The moderate to vigorous physical activity occupied around 5% of the time. There were no seasonal differences in the time spent at different intensities of physical activity. **b** shows the patterns for percentage of moderate to vigorous physical activity level

We divided the PA into four intensity categories using the cut-points defined by Freedson Adult VM3 2011 [44] which included sedentary to light (0 ~ 2689 CPM), moderate (2690 ~ 6166 CPM), vigorous (6167 ~ 9642 CPM) and very vigorous (>9643 CPM). We then quantified the percentage duration of PA at each activity level over the entire time each individual spent wearing the devices (Fig. 4a). Sedentary to light physical activity occupied around 95% of total time, the moderate to vigorous physical activity (MVPA), which included moderate, vigorous and very vigorous physical activity was less than 5% of the total time. Despite this MVPA was still on average around 504 (60*24*7*0.05) minutes per week. Month, temperature and PM2.5 were not significant factors associated with sedentary to light, moderate and very vigorous PA levels, but again ID was significant (Sedentary to light: F_(5,153)month_ = 1.05, *P* = 0.388; F_(33,153)ID_ =10.1, *P* < 0.001,F_(1,153)__temperature_ = 0.53, *P* = 0.469, F_(1,153)PM2.5_ = 0.31, *P* = 0.579; Moderate: F_(5,153)month_ = 0.79, *P* = 0.555; F_(33,153)ID_ =6.72, *P* < 0.001, F_(1,153)temperature_ = 0.69, *P* = 0.407, F_(1,153)PM2.5_ = 0.04, *P* = 0.837; Very Vigorous: F_(5,153)month_ = 1.22, *P* = 0.304; F_(33,153)ID_ = 1.91, *P* = 0.005, F_(1,153)temperature_ = 1.06, *P* = 0.305, F_(1,153)PM2.5_ = 0.01, *P* = 0.907). However, both month and ID were significantly associated with levels of vigorous PA. Temperature and PM2.5 were again not significant (Vigorous: F _(5,153) month_ = 3.15, *P* = 0.01; F_(33,153)ID_ =10.1, *P* < 0.001, F _(1,153)__temperature_ = 0, *P* = 0.995, F _(1,153)__PM2.5_ = 2.78, *P* = 0.098). Although there was an association between time spent in vigorous physical activity and month, it only occupied less than 0.5% of the time and this effect was insufficient to change the total physical activity level (Fig. 4b).

## Discussion

Physical inactivity has been identified as the fourth leading risk factor for global mortality causing an estimated 3.2 million deaths [1]. PA is thought to be largely under voluntary control and may therefore be highly responsive to various environmental factors – such as weather conditions, ambient temperature and air pollution levels. Since such factors vary spatially and temporally, the resultant variation in PA may contribute to the spatial and temporal variation in reported levels of non-communicable diseases [12].

Current studies on seasonal variation in PA have generated inconsistent results. Although most of the studies in European and North American populations [14, 33–37, 39–41] found that adults and children were generally more active in summer/spring and had lower PA levels in winter, using both self-reported measures and objective measurements. A study in Australia found that children were more active in winter rather than summer [43]. Studies in adolescents suggested that that there was no seasonal variation in physical activity [42].

To date no studies of seasonal variation have been performed in Han Chinese populations. In our study we found that there was no seasonal differences in the average hourly PA, despite the fact that there were large significant differences in ambient temperature and PM2.5 across the seasons and time of day. In summer in Beijing it was on average 25 °C hotter than in winter, during these measurements, while the air-quality was much worse in winter than in the other seasons [21]. A potential explanation for the absence of a seasonal effect on PA is that the weeks we measured were all working weeks, and the subjects we measured, who were all in employment, adopted a daily activity pattern that was largely dominated by the demands of their work and unresponsive to external stimuli. This finding was consistent with several previous publications [45, 46] that found sedentary to light physical activity occupied almost all of the total physical activity (using Freedson VM2011). In our study, the sedentary to light physical activity occupied over 95% of the time and there was no seasonal difference of sedentary to light, moderate and very vigorous physical activity. Although we found a seasonal effect on vigorous physical activity, it occupied less than 0.5% of the time. Such high levels of sedentariness may explain why there was no seasonal difference of overall physical activity. MVPA is regarded as a factor that influences the health status and a suggested 150 min per week would provide benefits to health outcomes. In our work, the MVPA level was almost 5% of total physical activity which is equivalent to 500 min per week. This indicates that all these subjects met the general recommendation of physical activity of the world health organization.

Previous studies, across a range of age groups from children to the elderly, including studies that have used the same activity logger we used, have suggested that PA levels differ between the sexes [43, 45, 47, 48]. This was consistent with our results where we found males were more active than females. Age and body fatness have also been suggested to be important correlates of physical activity. In contrast to several previous studies that indicated activity declines in older people [46, 47, 49] we found a positive relationship with age: older individuals in our sample were more active than the younger volunteers. It is important to note that because of the cross sectional nature of our study this does not imply that individuals in Beijing become more active as they get older, only that individuals born longer ago are currently more active. A number of previous studies [50–53] have shown a negative correlation between physical activity and body fatness. Recent studies have suggested this is likely because increasing levels of obesity suppress activity rather than the reverse [54, 55]. Our study also showed the similar results. The EPIC-Norfolk study [56] also suggested a physically active lifestyle is associated with 40% reduction in genetic predisposition of common obesity.

Time of day was the most significant factor influencing the hourly PA levels. This was partly because of the strong diurnal pattern in activity with most people asleep and inactive between midnight and 6:00 am. Nevertheless, if we excluded the main sleeping time from the analysis, time of day still remained the strongest factor. We found that the hour with the greatest physical activity in all the months except November was 18:00 to 19:00. In November it was 17:00 to 18:00. The consistent timing of this early evening activity throughout the year was probably driven by the invariant nature of work patterns independent of season. This potentially explains why there were also no significant effects of ambient temperature and PM2.5 as individuals timed their activities according to their work days rather than responding to environmental cues. It is worth knowing that all the volunteers we recruited had occupations where they worked indoors. Patterns may be different for individuals with occupations that include significant levels of outdoor activity. Nevertheless although the peak evening activity coincided with the likely commuting time, these individuals remained relatively active throughout the evening suggesting that also the individuals had rather invariant patterns of leisure time activity as well.

The current study indicated that a single week long measurement by the GT3x would be representative of physical activity levels of this population without serious seasonal bias. One limitation of our study is the GT3x couldn’t measure the physical activity under water such as swimming and upper body movements during activities like cycling. This may underestimate the exact physical activity of our subjects, although according the life-style questionnaire with our subjects, few of them chose to go swimming. Another limitation of this longitudinal study was the relatively small sample size of subjects that completed the entire study. It was very hard to keep tracking the same people over the entire year and the dropout rate was high due to various reasons like pregnancy and changing jobs – hence moving out of the area. It would also have been useful for the subjects to have completed a more detailed activity diary during each measurement period so that we knew not only how active they had been but what they were actually doing.

## Conclusions

In our study, we found that there was no seasonal variation in physical activity and no significant effects of ambient temperature and air pollution (PM2.5). People’s PA level was most responsive to time of day rather than other factors, presumably because individuals followed a fairly constant behavior pattern driven by their work demands. Despite this individual characteristics including body fatness, sex and age also influenced the PA level. Older individuals and males were more active. For surveys of activity our data indicate that measurements on a single week long occasion would be sufficient to represent the physical activity level of this population.

## Additional file

Additional file 1: Human subject criterion file Life-style questionnaire (in Chinese). **Figure S1.** Workflow of the experiment. 40 subjects were recruited at the beginning of the experiment. Basic demographic information was captured. They came to visit lab every two months to get the accelerometer GT3X fitted and measured the body composition. (DOCX 205 kb)

## Abbreviations

BMI: Body mass index
CPM: Counts per minute
GLM: General linear model
MVPA: Moderate to vigorous physical activity
PA: Physical activity
PM2.5: Particulate matter with a diameter less than 2.5 microns
VM: Vector magnitude
