# Supplementary material for: No seasonal variation in physical activity of Han Chinese living in Beijing
Source: Int J Behav Nutr Phys Act. 2017 Apr 17;14:48. doi: 10.1186/s12966-017-0503-1 (PMC5392911; doi:10.1186/s12966-017-0503-1)
Supplement: Additional file 1: — Human subject criterion file Life-style questionnaire (in Chinese). Figure S1. Workflow of the experiment. 40 subjects were recruited at the beginning of the experiment. Basic demographic information was captured. They came to visit lab every two months to get the accelerometer GT3X fitted and measured the body composition. (DOCX 205 kb) [file 12966_2017_503_MOESM1_ESM.docx]

**Additional Information**

Additional Figure S1

Human subject criterion file

Life-style questionnaire (in Chinese)

**Additional Figure**

**Figure S1** Workflow of the experiment. 40 subjects were recruited at the beginning of the experiment. Basic demographic information were measurement. They came to visit lab every two months to get the accelerometer GT3X and measured the body composition.


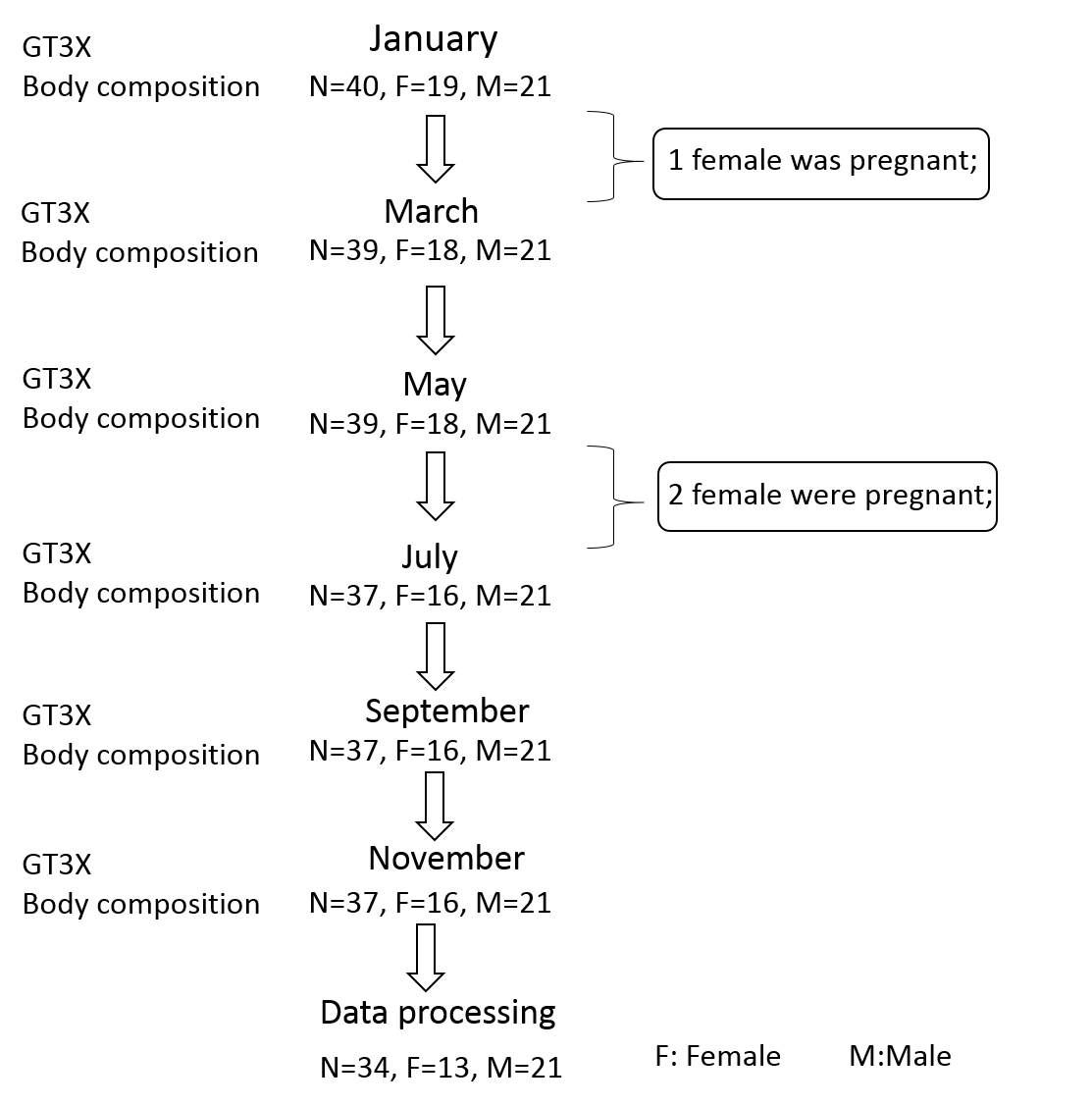


**Human subject criterion file**

**Project Name:**  Seasonal Variability in Physical Activity of Han Chinese living in Beijing

**Aims**: There are two aims of this study

a) To guide future studies of number of measurements needed to represent activity patterns

b) To explore environment factors (temperature, and air quality) that impact on physical activity in Han Chinese in Beijing throughout the year

**Subjects:** We aim to recruit 40 adult Beijing urban citizens who will live in Beijing for one year

**Inclusion criteria:**

- - Age:20-55
  - Will be in Beijing throughout the year

**Exclusion criteria:**

- - Disabled
  - Others who can’t do any physical activity
  - Pregnant(or planning to throughout the year)
  - Lactation (or planning to throughout the year)
  - Non-Han Chinese

**Recruiting method**

- - Recruitment posters near the University of Chinese Academy of Science Campus and Olympic campus of Chinese Academy of science

**Methodology-GT3X measurement**

- **Step1: Before measurement:（2014.12）**
  - - Basic information
    - Informed consent
    - Life-style Questionnaire
- **Step2: Measurement（2015.01-2015.12）**
  - - **Wearing activity logger GT3X（2015.01）**
    - 2015.01： get GT3X, measure basic body parameters(including height, weight, waist circumference, hip circumference, heart rate, blood pressure), body composition using TANITA, spirometry
    - **Wearing activity logger GT3X（2015.03）**
    - 2015.03：get GT3X, body composition
    - **Wearing activity logger GT3X（2015.05）**
    - 2015.05：get GT3X, body composition
    - **Wearing activity logger GT3X（2015.07）**
    - 2015.07：get GT3X, body composition
    - **Wearing activity logger GT3X（2015.09）**
    - 2015.09：get GT3X, body composition
    - **Wearing activity logger GT3X（2015.11）**
    - 2015.11：get GT3X, body composition

**Calendar（we aim to begin at 12/2014 and end in 12/2015）**

| **Week 0**  **（2014/12）** | **Week 1**  **（2015/01）** | **Week 1**  **（2015/01）** |
| --- | --- | --- |
| - Basic information - Informed consent - Life-style Questionnaire | Wearing activity logger GT3X | - get GT3X back - body composition |
|  | **Week 2**  **（2015/03）** | **Week 2**  **（2015/03）** |
|  | Wearing activity logger GT3X | - get GT3X back - body composition |
|  | **Week 3**  **（2015/05）** | **Week 3**  **（2015/05）** |
|  | Wearing activity logger GT3X | - get GT3X back - body composition |
|  | **Week 4**  **（2015/07）** | **Week 4**  **（2015/07）** |
|  | Wearing activity logger GT3X | - get GT3X back - body composition |
|  | **Week 5**  **（2015/09）** | **Week 5**  **（2015/09）** |
|  | Wearing activity logger GT3X | - get GT3X back - body composition |
|  | **Week 6**  **（2015/11）** | **Week 6**  **（2015/11）** |
|  | Wearing activity logger GT3X | - get GT3X back - body composition |

**Life style questionnaire**

**生活方式问卷调查**

编号

问卷调查完成的时间_______年_______月_______日

**本次问卷调查的目的是评估您的某些生活方式与您身体活跃性之间的关系。问题包括您拥有或者可供您使用的一些设施，以及您平时的某些生活习惯。本次问卷有21个问题，只需10分钟即可完成。请您尽可能完整的回答以下问题。本问卷的所有信息将以匿名编号的形式保存，不会涉及您的个人信息。**

1. 您的家里是否有以下项目？如果您拥有、租用或者在以其他方式使用以下项目，请在相应的选项后面打勾：

自行车 □ 洗衣机 □ 健身车 □ 台式电脑 □

电动车 □ 洗碗机 □ 跑步机 □ 笔记本电脑 □

摩托车 □ 滚筒式烘衣机 □ 轮滑　　　　□ 平板电脑（如iPad）□

汽车 □ 微波炉 □ 智能手机 □ 游戏机 □

1. 您的家里有几台（或者在租用）电视机？ ____________台

您的卧室里是否有电视机？ 是 □ 否 □

您每天大约花几个**小时**时间看电视？ ____________小时

1. 如果您有游戏机／电脑／智能手机／iPad的话，您每天花多长时间用它们来从事以下娱乐活动？

玩游戏 ____________分钟

上网冲浪 ____________分钟

看视频、电影或电视剧 ____________分钟

1. 您的职业是？（如果已退休请注明） ________________

您认为您工作中体力劳动强度有多大？ ________________（请用具体的数字0~10表示）

参考评价标准：0、1、2、3 大部分工作时间坐着

4、5、6 大部分时间站立

7、8、9、10 站立且需要重体力劳动

1. 您家距离工作单位多远？请选择最接近的答案：

1千米（1公里）之内□ 1 到5 千米□ 5 到10 千米□ 超过10千米□

不适用 □

1. 您通常采用什么交通方式上下班/上下学？

步行 □ 公交车或班车(加步行) □

骑自行车 □ 地铁 (加步行) □

骑电动车或摩托车 □ 打车 □

开车 □ 搭乘他人的自行车/电动车/摩托车 □

其他 □ 请注明具体方式________________

不适用 □

1. 如果您平时乘坐公交车或地铁上下班/上下学，并步行去公交车站或地铁站，请注明您

每天共需要步行多长时间。 _____________ 分钟 不适用 □

1. 您通常上下班/上下学需要多长时间？ _____________ 分钟 不适用 □
2. 您通常几点去上班/上学？ _______________ 不适用 □

1. 您通常几点下班/放学回到家? _______________ 不适用 □
2. **在过去的7天里，您有几天在上班**? _______________天
3. 您工作日通常几点起床？ _______________

您**非工作日**通常几点起床？ _______________

1. 您工作日通常几点睡觉？ _______________

您**非工作日**通常几点睡觉？ _______________

1. **在过去的7天里**，您是否参与过以下运动？请在您参与过的运动后打勾，并请注明在过去7天里您大概运动了多长时间。（也包括您参加某项运动训练）

篮球 □ ___________ 分钟/每天

足球 □ ___________ 分钟/每天

乒乓球 □ ___________ 分钟/每天

羽毛球 □ ___________ 分钟/每天

排球 □ ___________ 分钟/每天

网球 □ ___________ 分钟/每天

高尔夫 □ ___________ 分钟/每天

游泳 □ ___________ 分钟/每天

跑步 □ ___________ 分钟/每天

柔道 □ ___________ 分钟/每天

空手道 □ ___________ 分钟/每天

跆拳道 □ ___________ 分钟/每天

太极拳 □ ___________ 分钟/每天

公共健身器材 □ ___________ 分钟/每天

其他  □ 请注明具体的运动项目______________

时间： ______________ 分钟/每天

无 □

1. 您是否是某个运动会所或者俱乐部的会员？ 是 □ 否 □

如选择是，**在过去的7天里**，您是否去过俱乐部做运动？ 是 □ 否 □

如选择是，您在那里花了多长时间做运动？ ______________ 分钟/每天

1. **在过去的7天里，**您外出去过几次以下公共场所？请您在去过的场所选项后打勾并注明您总共大概去了几次一下几个场所。

电影院 □ ____________次/周

餐馆 □ ____________次/周

酒吧 □ ____________次/周

KTV □ ____________次/周

迪厅 □ ____________次/周

其他 □ 请注明具体的场所类型______________

次数： ______________ 次/周

无 □

1. **在过去的7天里，**您是否参加过以下活动？请您在参加过的活动后打勾并注明您总共大概活动了多长时间。

户外徒步 □ ___________ 分钟/每天

外出购物 □ ___________ 分钟/每天

钓鱼  □ ___________ 分钟/每天

放风筝 □ ___________ 分钟/每天

踢毽子 □ ___________ 分钟/每天

柔力球 □ ___________ 分钟/每天

中国传统舞（包括秧歌） □ ___________ 分钟/每天

夜店跳舞 □ ___________ 分钟/每天

古典舞(交谊舞, 芭蕾等) □ ___________ 分钟/每天

其他 □ 请注明具体的运动项目______________

时间： ______________ 分钟/每天

无 □

1. 假设您要从某个大楼的楼底去三楼找人。通常情况下，您会首先选择电梯还是楼梯？

电梯 □ 楼梯 □

1. 您是否吸烟？ 是 □ 否 □

如选择是，您通常每天吸多少支烟？ 中国烟 __________支

进口烟 __________支

1. 您如何评价自己日常生活的活跃程度？ ___________（请用**具体的**数字0~10表示）

评价标准参考：0、1、2、3表示很少运动；

4、5、6表示中等运动频率，有时候做运动；

7、8、9、10表示经常做运动

1. 您的婚姻状况？

不想回答 □ 单身 □ 恋爱或者同居关系中 □ 已婚 □

如果您选择已婚或在恋爱中，您如何评价**您的伴侣**生活方式的活跃程度？

___________（请用具体的数字0~10表示；评价标准与第20题相同。）
